# Timeline of the Development of Skin-Lightening Active Ingredients in Japan

**DOI:** 10.3390/molecules27154774

**Published:** 2022-07-26

**Authors:** Kazuhisa Maeda

**Affiliations:** School of Bioscience and Biotechnology, Tokyo University of Technology, 1404-1 Katakura, Hachioji, Tokyo 192-0982, Japan; kmaeda@stf.teu.ac.jp; Tel.: +81-426-372-442

**Keywords:** skin-lightening, pharmaceutical cosmetics, quasi-drug, ingredient, melasma, pigment spots

## Abstract

Japanese pharmaceutical cosmetics, often referred to as quasi-drugs, contain skin-lightening active ingredients formulated to prevent sun-induced pigment spots and freckles. Their mechanisms of action include suppressing melanin production in melanocytes and promoting epidermal growth to eliminate melanin more rapidly. For example, arbutin and rucinol are representative skin-lightening active ingredients that inhibit melanin production, and disodium adenosine monophosphate and dexpanthenol are skin-lightening active ingredients that inhibit melanin accumulation in the epidermis. In contrast, oral administration of vitamin C and tranexamic acid in pharmaceutical products can lighten freckles and melasma, and these products are more effective than quasi-drugs. On the basis of their clinical effectiveness, skin-lightening active ingredients can be divided into four categories according to their effectiveness and adverse effects. This review discusses academic research and development regarding skin-lightening ingredients in Japan.

## 1. Development of Skin-Lightening Active Ingredients

Japanese pharmaceutical cosmetics are required to have one of the following purposes of use: (1) cleansing, (2) beautifying, (3) increasing attractiveness, (4) changing appearance, and (5) maintaining healthy skin or hair. Whether or not the product has the above purpose of use should be clarified by the efficacy or effectiveness, usage, and dosage of the product. If a product is determined not to meet the specified purpose of use on these bases of its efficacy or effectiveness, usage, and dosage, it is considered a “drug.” Examples of indications include “spots, freckles, pigmentation due to sunburn, etc.” (internal use), “spots, freckles, pigmentation due to sunburn/rash” (internal use), “skin pigmentation, senile pigmentation” (external use), “spots” (internal use), “Riehl melanosis, post-inflammatory hyperpigmentation” (for injection), and “melasma, freckles, post-inflammatory hyperpigmentation” (for internal use and injection). If the product has only the above efficacy or effectiveness, usage, and dosage, it cannot be considered a pharmaceutical cosmetic (quasi-drug).

In Japan, the efficacy for pharmaceutical cosmetics was changed in 2019 from “prevents sun spots and freckles” to “prevents sun spots and freckles by suppressing melanin production.” In 2004, the action of the ingredients in the formulation was changed to “prevents spots and freckles,” and, on the basis of the mechanism of action of the ingredients in the formula, the indication of “suppressing the accumulation of melanin and preventing spots and freckles” was approved. Thus, it is possible to apply for approval of pharmaceutical cosmetics for new efficacy within the scope of quasi-drug efficacy, on the basis of a clear scientific rationale.

**Figure 1:**
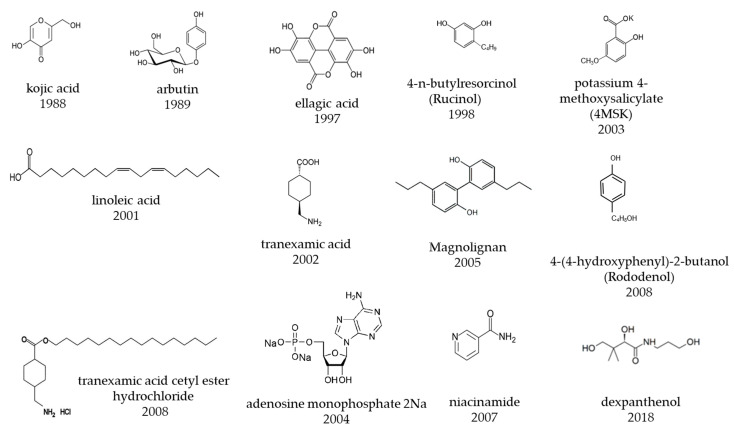
Chemical structure of skin-lightening active ingredients in Japan.

The Japanese skincare market can be divided into the following functional categories: moisturizing, skin-lightening, anti-aging, sensitive skin, and pore and acne care. Lightening of the skin accounts for approximately 30% of the market and is the category with the most rapid growth over the past 35 years, particularly in terms of research and development [1]. Approximately twenty active ingredients have been developed for lightening-related quasi-drugs, including chemical substances and plant extracts with excellent inhibitory effects on tyrosinase activity and melanin production. A chronology of the development of skin-lightening active ingredients is presented in Table 1. The chemical structures of skin-lightening active ingredients in Japan are presented in Figure 1. Recent research on anti-aging skin-lightening has revealed new mechanisms and methods for the treatment of skin lightening. This section introduces the history of the skin-lightening ingredients developed in Japan.

### 1.1. Ascorbic Acid Derivatives

**Figure 2:**
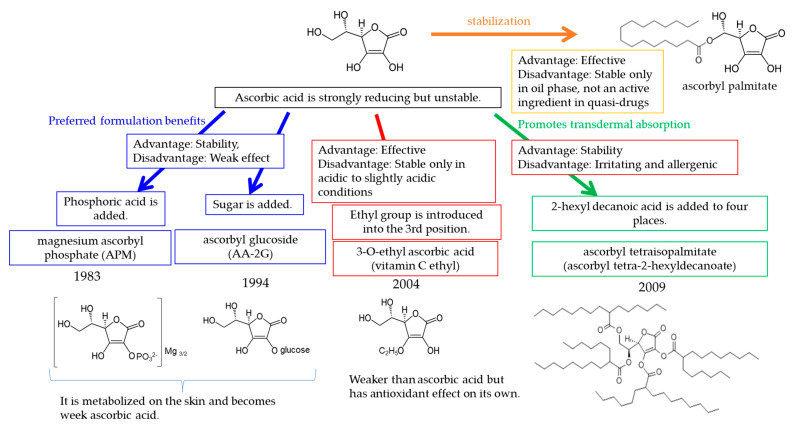
Vitamin C derivatives in skin-lightening ingredients in Japan.

Figure 2 shows the developmental chronology of ascorbic acid derivatives used in Japanese skin-lightening pharmaceutical cosmetics. The ascorbic acid derivative magnesium L-ascorbyl-2-phosphate (magnesium ascorbyl phosphate; APM) was developed as the first skin-lightening ingredient more than 40 years ago. Because ascorbic acid easily discolors over time when used in formulations, thus decreasing its content, more stable ascorbic acid fatty acid esters need to be developed, such as such as ascorbyl stearate or ascorbyl palmitate. However, even these fatty acid esters are not sufficiently stable in formulations containing water, such as emulsions; thus, browning occurs. A more stable APM was developed by phosphorylation of the hydroxyl group at position 2 of ascorbic acid to form magnesium salt; this compound was approved for use in Japanese quasi-drugs in the 1980s. APM has been used for the treatment of hyperpigmentation (melasma and Riehl melanosis) since 1969 [2], and its effectiveness in the treatment of sunburn-induced hyperpigmentation has been demonstrated in well-controlled studies since 1983 [3,4]. To make APM more stable under weakly acidic conditions, glucose has been attached to the hydroxyl group at position 2 to form 2-O-α-D-glucopyranosyl-L-ascorbic acid (ascorbyl glucoside; AA-2G), a compound that is stable around pH 6 that was approved as an active ingredient in quasi-drugs in the 1990s [5,6]. Unlike AA-2G, 3-O-ethyl-L-ascorbic acid (3-O-ethyl ascorbic acid; vitamin C ethyl), a vitamin C derivative with antioxidant and skin-lightening effects, was developed and approved as an active ingredient in quasi-drugs in 2004 [7,8]. Although ultraviolet A radiation contributes to persistent pigmentation [9], and DHICA polymerization is involved in the mechanism [10], vitamin C ethyl inhibits both polymerization of DHICA and persistent pigmentation formation [7]. 3-O-ethyl ascorbic acid manufactured by Nippon Hypox Laboratories Inc. is not allergenic, and no evidence of induced allergic contact dermatitis was recorded during a study by the National Industrial Chemicals Notification and Assessment Scheme (NICNAS) [11]. However, allergic contact dermatitis has been reported for other manufactured ingredients [12]. This may be due to impurities in other manufacturing materials. AA-2G is stabilized by modification of the hydroxyl group at position 2, whereas 3-O-ethyl ascorbic acid differs in that the ethyl group is ether-linked to the hydroxyl group at position 3. The fat-soluble vitamin C derivative ascorbyl tetra-2-hexyldecanoate (ascorbyl tetraisopalmitate) has also been developed [13], but it has been associated with allergic contact dermatitis [14,15,16].

### 1.2. Placenta Extract

Placenta extract, which dates back to ancient Egypt, is used mainly in cosmetic medicine for injections and internal administration. Placenta has been used for more than 60 years since its approval as a pharmaceutical injection in 1956 in Japan. It has been used as an OTC drug for frostbite and chapped skin and, for the purpose of enhancing cell division and promoting metabolism. The use of 3% human placenta extract preparation for treating facial hyperpigmentation in women was reported in 1982 in 47 cases of melasma, and two cases of freckles [17,18,19].

Placental extract used in pharmaceutical cosmetics is obtained by aseptic extraction of water from the placenta of pigs [*Sus scrofa domest*icus Erxleben (*Suidae*)] by freezing, thawing, or other methods, and removal of the high molecular weight fraction containing 0.01–0.40% nitrogen [placenta extract (1)] or 0.02–0.15% of nitrogen, as well as the macromolecular fraction containing 100 KAU or more of alkaline phosphatase [placenta extract (2)]. In addition, an extract is obtained by aseptic extraction of water from the placenta of pigs by enzymolysis or other methods, thus yielding a fraction containing 0.15–0.48% nitrogen [placenta extract (3)]. Common constituents include amino acids; vitamins such as ascorbic acid, thiamine, pyridoxine, and niacin; nucleic acid components such as uracil, adenine, and guanine; carbohydrates; lipids; and minerals. 

Placenta extract promotes tissue metabolism and inhibits tyrosinase activity [20]. Moreover, the protein/peptide and lipid fractions of human placenta have melanogenesis-promoting effects [21,22]. Inhibitory or promoting effects on melanogenesis have been observed, depending on the extracted biomolecules, and the mechanisms of action are still largely unknown.

### 1.3. Kojic Acid

Kojic acid (5-hydroxy-2-hydroxymethyl-4-pyrone) is a skin-lightening ingredient obtained by cultivating yeast used in the production of soy sauce and miso. Topical application of a 1% or 2.5% kojic acid formulation has been shown to be effective in treating senile pigmentation and melasma [23,24,25,26], and kojic acid was approved as an active ingredient in quasi-drugs in 1988. In 2003, however, oral administration of kojic acid to p53-deficient mice was reported to induce liver cancer [27]. The Ministry of Health, Labour and Welfare of Japan suspended its use from March 2003, pending further research to clarify whether kojic acid is carcinogenic or genotoxic. A cosmetics manufacturer has conducted additional tests on the cosmetic properties of kojic acid and found it to be safe [28]. Accordingly, the Ministry of Health, Labour and Welfare issued an opinion at the Subcommittee on Safety Measures for Medicinal Products of the Pharmaceutical Affairs and Food Sanction Council on 2 November 2005, stating: “When used appropriately in quasi-drugs, there should be no particular concern about safety”. Subsequently, the aforementioned notice of discontinuance of use was withdrawn, and the resumption of production and marketing of quasi-drugs containing kojic acid was approved. However, simultaneous administration of ascorbic acid has been reported to enhance the liver tumor-promoting effect of kojic acid in rats induced with diethyl nitrosamine [29].

In a human transdermal absorption study in which 500 mg of cream containing 1% kojic acid was applied daily to the faces of six healthy women, the plasma concentration (limit of quantification 1 ng/mL) was highest 3–6 h after application, and the mean maximum plasma concentration in the six participants was 1.54 ng/mL. The highest plasma concentration averaged 1.54 ng/mL in the six participants. After more than 10 years of experience in using kojic acid in humans, no adverse health effects have been reported [30] Thus, the risk of carcinogenicity is extremely low for concentrations of kojic acid below 3%, and kojic acid is unlikely to exhibit genotoxicity that would harm living organisms. Furthermore, because kojic acid at a 1% concentration is unlikely to be absorbed into the body through the skin, and no adverse health effects have been reported, the safety of kojic acid is not of concern in cosmetics and pharmaceutical cosmetics under normal conditions of use. Products containing kojic acid are being sold as before.

### 1.4. Hydroquinone Derivatives and Phenolic Compounds

Focusing on the hydroquinone and the phenolic hydroxyl group of kojic acid, a hydroquinone derivative and a lightening agent with a phenolic hydroxyl group have been developed that have a stronger inhibitory effect on melanogenesis than kojic acid. Arbutin (hydroquinone β-D-glucopyranoside) was developed and approved as an active ingredient in quasi-drugs in 1989, on the basis of clinical results of its effectiveness against melasma [31]. Arbutin, a hydroquinone glycoside, is found in the leaves of *Arctostaphylos uva ursi*, with a content of 5–7.5%, as listed in the Japanese Pharmacopoeia. Arbutin inhibits melanogenesis at concentrations that do not cause cytotoxicity to pigment cells through antagonistic inhibition of tyrosinase activity [32,33]. Arbutin inhibits tyrosinase activity in a reversible manner and therefore is safer than the nonspecific or irreversible inhibition mediated by other tyrosinase inhibitors [32,33]. A variety of active ingredients have been developed to control tyrosinase activity in cosmetics since then, including ellagic acid [34,35,36], 4-n-butylresorcinol (Rucinol) [37,38,39,40], 5,5′-dipropyl-biphenyl-2,2′-diol (Magnolignan) [41,42,43], 4-(4-hydroxyphenyl)-2-butanol (Rhododenol) [44], and potassium 4-methoxysalicylate. Ellagic acid is a natural phenolic antioxidant, whereas the other five components are chemically synthesized. Magnolignan inhibits tyrosinase maturation, and cases of allergic contact dermatitis have been reported [45]. Rhododenol interferes with several steps of melanin production, including inhibiting tyrosinase activity, accelerating tyrosinase degradation, and targeting tyrosinase-related proteins, thus decreasing eumelanin levels. Rhododenol and Magnolignan both release hydroxyl radicals in the presence of tyrosinase in melanosomes and therefore are cytotoxic to pigmented cells [46,47]. Rhododenol is no longer used in pharmaceutical cosmetics, because of its leukoderma-inducing properties. Magnolignan is also no longer included in pharmaceutical cosmetics. Consequently, a 1-year safety review of all new quasi-drugs used in skin-lightening products is required before their approval.

### 1.5. Natural Compounds Contained in Plants

Research and development regarding natural compounds contained in plants and cosmetic raw materials to inhibit melanin production has been actively pursued, and many plant extracts have been incorporated into pharmaceutical skin-lightening cosmetics as well as quasi-drugs. For example, chamomile (*Matricaria chamomilla* L.) extract is an active ingredient in quasi-drugs that inhibits the action of endothelin [48,49,50,51], and safflower oil contains a high content of linoleic acid [52,53,54,55], which has been reported to promote both epidermal turnover and tyrosinase degradation [53,55]. The extract of *Arnica montana*, which is used as a cosmetic ingredient in Japan, has been found to inhibit melanogenesis; the active ingredient has been identified as a traxastane-type triterpene [56]. In pigmented cells, suppression of the expression of tyrosinase, tyrosinase-related protein-1 (Tyrp1), tyrosinase-related protein-2 (Tyrp2), and Pmel17 has been proposed to be the mechanism underlying melanogenesis inhibition [56]. Scutellarein, a flavonoid found in the medicinal plant *Scutellaria baicalensis* Georgi, inhibits melanin production by suppressing tyrosinase activity and microphthalmia-associated transcription factor, a protein required for the development of melanocytes [57].

### 1.6. Tanexamic Acid, Adenosine Monophosphate and Dexpantenol

Skin-lightening ingredients that act on keratinocytes without acting on melanocytes include tranexamic acid, adenosine monophosphate, and dexpanthenol.

In the 2000s, comprehensive skin-lightening research efforts focused on the various processes involved in melanogenesis, as opposed to only direct inhibition of melanogenesis. Phospholipase A2, arachidonic acid metabolites, and histamine were found to promote melanin synthesis [58,59,60], and tranexamic acid was found to be effective against ultraviolet B (UVB)-induced skin redness and pigmentation in guinea pigs [61]. Oral tranexamic acid was found to be effective for melasma [62] and was marketed as an over-the-counter option for treating melasma in Japan in 2007. Several other studies have demonstrated the effectiveness of tranexamic acid in oral administration [63,64,65] as well as its utility as a topical agent [66,67,68,69]. The skin-lightening effects of tranexamic acid are believed to result from both prostaglandin (PG) production mediated by plasmin [70,71] and cytokine production [72]. As of 2008, tranexamic acid cetyl ester hydrochloride (TXC), which is tranexamic acid esterified with cetyl alcohol, was also approved as an active ingredient [73,74]. TXC is hydrolyzed by an esterase and is likely to be converted to tranexamic acid in the subepidermal layer. Because both TXC and tranexamic acid suppress PGE2 production, TXC has been suggested to inhibit melanogenesis by preventing the production of melanocyte activating factors, particularly PGE2 production. Disodium adenosine phosphate was also developed as an active ingredient to suppress melanin accumulation by promoting epidermal turnover [75]. Furthermore, vitamin B3, niacinamide, has been reported to block the transfer of melanosomes at the surfaces of epidermal cells [76,77,78,79]. Some of these skin-lightening ingredients are also used in Korea and China. Another provitamin B5 called dexpanthenol was also approved in 2018 for use as a quasi-drug to decrease melanin accumulation and prevent spots and freckles, because of its ability to convert metabolites into energy and promote the turnover of epidermal cells [80,81].

**Figure 3:**
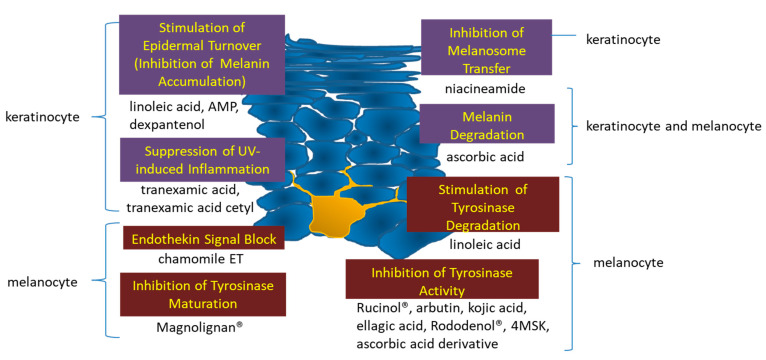
Point of action of skin-lightening active ingredients on the epidermis.

Figure 3 illustrates their mechanisms of action. Since approximately 40 years ago, technological development has progressed from active ingredients that act on tyrosinase to those that act on tyrosinase genes, melanocytes, and the epidermis, thus leading to a wide variety of active ingredients and mechanisms of action in current formulations. Research on the delivery of active ingredients in skin-lightening products is also progressing. In contrast to sunburn pigment, which fades spontaneously as melanin is expelled during epidermal cell turnover, senile pigmentation does not fade spontaneously but instead worsens and progresses gradually, thereby suggesting a different mechanism from that in sunburn. Technological advances will continue to produce new active ingredients with high effectiveness in skin-lightening products in the future.

## 2. Report on the Effectiveness of a Formulation Containing a Lightening Agent and a Spot Remedy for Senile Pigmented Lesions

Clinical studies on senile pigmentation have investigated a formulation containing magnesium ascorbate phosphate salt, arbutin, and ellagic acid, which are active ingredients in quasi-drugs that prevent melanin production and freckles; a formulation containing kojic acid, which is no longer used in quasi-drugs because of concerns over its carcinogenic properties; a formulation containing vitamin A acid [82,83,84], which is involved in regulating epidermal turnover and is used in the United States to treat diseases such as acne vulgaris and psoriasis, and is effective in treating wrinkles and pigmentation caused by sun damage; and a formulation containing 2% 4-hydroxyanisole and 0.01% vitamin A acid, which is marketed in the United States as a treatment for senile pigmentation [85,86]. The effectiveness of these compounds is summarized in Table 2.

In an open study of 34 patients, including 17 with senile pigmentation, patients were given a formulation containing 10% magnesium L-ascorbyl-2-phosphate—a content exceeding the 3% in most quasi-drugs. A total of 88.3% of patients with senile pigmentation showed slight or even higher effectiveness, as assessed by skin color measurement [4]. A 7% arbutin formulation demonstrated an effectiveness rate of 81.3% on senile pigmentation after 3 months in an open test, and effectiveness was observed for all patients when the treatment period was extended to 6 months and 1 year [87]. An open study in 13 women treated with a 0.5% formulation of ellagic acid for 1–3 months found improved effectiveness on senile pigmentation in 69.2% of participants [36]. In a study in 18 patients (5 men and 13 women) with senile pigmentation who received topical application of 1% kojic acid and 0.1% oil-soluble licorice extract over a 16-week period, 77.8% of participants showed an improved effectiveness rate [26]. Additionally, 2% 4-hydroxyanisole and 0.01% vitamin A acid have been found to be more effective than 3% hydroquinone in treating senile pigmentation [85]. In a double-blind study investigating the topical effectiveness of 2% 4-hydroxyanisole for 6 months in 421 patients, the improved effectiveness was 84.1%. Complete disappearance of pigmented lesions was observed in some cases, thus indicating high effectiveness, but some adverse effects including erythema and irritation were also observed [86].

Reported effectiveness rates differ depending on whether the effectiveness criteria were “effective”, “somewhat effective”, higher. In each study, different criteria are used to determine the level of improvement as “effective” or “somewhat effective”. In some studies, the criteria for determining effectiveness are unclear, and placebos may or may not be used to control effectiveness. Therefore, caution should be exercised in comparing effectiveness rates across studies in which the criteria for determining effectiveness are unclear or in open studies lacking placebo controls.

**Table 2:** Comparison of clinical trials of skin-lightening agents and drugs for the treatment of age spots.

		10% Magnesium Ascorbyl Phosphate Formulation	7% Arbutin Formulation	0.5% Ellagic Acid Formulation	1% Kojic Acid and 0.1% Oil-Soluble Licorice Extract Formulation	2% 4-Hydroxyanisole and 0.01% Vitamin A Acid Formulation	
Test design		Open study	Open study	Open study	Open study	Double-blind controlled study	
Number of cases		17	16	13	18	420	421
Sex		–	–	Women	5 men, 13 women	Men and women	
Age		–	–	Unknown	28–50 years old (average 39 years old)	34–85 years old (average 62.6 years old)	
Location		–	–	Unknown	Face	Forearm	Face
Period		–	3 months to 1 year	1 to 3 months	8 and 16 weeks	24 weeks	Observation up to 48 weeks after 24 weeks of application
Effectiveness judgment		Skin color value (color difference meter)	Visual observation	Visual observation	Close-up photograph determination and skin color value (image analysis)	Visual observation	Visual observation
Effectiveness ratio	Effective (improved or much improved) or higher	58.80%	3 months 0%, 6 months 15.4%, 1 year 66.7%	30.80%	8 weeks 5.6%, 16 weeks 22.2%	52.60%	56.30%
	Slightly effective (slightly improved) or more	88.20%	3 months 81.2%, 6 months 100%, 1 year 100%	69.20%	8 weeks 66.7%, 16 weeks 77.8%	79.30%	84.10%
Adverse effects		–	None	None	Irritation in a few cases, but no serious adverse effects	Redness 56%, burning 34%, desquamation 24%, itching 16%, irritation 7%, decoloration 9%.	–
References		[4]	[87]	[36]	[26]	[86]	[86]

## 3. Effectiveness Indices of Lightening Ingredients Developed in Japan

Most Japanese tests of the effectiveness of pharmaceutical skin-lightening cosmetics have assessed the degree of prevention of skin darkening caused by artificial UVB irradiation. Skin-lightening effects are exclusively evaluated on skin that darkens when exposed to ultraviolet light in humans. There are two types of evaluation methods: the anti-pigmentation test and the accelerated pigmentation disappearance test. The former is a double-blind controlled study in which a test sample is applied before, during, or after the UV irradiation period and the degree of pigmentation formation is compared with a placebo. The latter is a double-blind controlled trial in which the test sample is applied to the pigmented area and the degree of fading of the pigmentation is compared with a placebo. Both are assessed by visual evaluation and instrumental measurement based on superiority or inferiority comparisons or score judgments.

Consumers expect that skin lightening products will “lighten spots and freckles”. Moreover, many consumers expect these products to “eliminate spots and freckles” [88] and want skin-lightening cosmetics to have an effect on spots that have already formed, rather than preventing formation, or promoting the fading, of pigmentation caused by ultraviolet rays. However, problems exist regarding the lightening effects of such cosmetics when actually used on spots and freckles: test results published in scientific journals are lacking, and a possibility exists that these cosmetics might cause skin problems.

Skin-lightening active ingredients can be divided into four categories according to their clinical effectiveness and adverse effects. This review discusses academic research and development regarding skin-lightening ingredients in Japan. We have compiled a table of effectiveness indices for Japanese skin-lightening ingredients, on the basis of the published scientific literature (Table 3).

Category A: Effectiveness of the same concentration of cosmetic formulations on human pigment spots has been described in scientific journals and is highly recommended. Examples include tranexamic acid, arbutin, 3-O-ethyl ascorbic acid, magnesium ascorbyl phosphate (APM), ellagic acid, kojic acid, linoleic acid, 4-n-butylresorcinol, chamomile extract, and adenosine monophosphate.

Category B: Effectiveness of higher concentrations than those used in cosmetics on human pigment spots has been described in scientific journals and is recommended. Examples include oil-soluble licorice extract containing 50% glabridin, niacinamide, placenta extract, retinol, ascorbyl glucoside (AA-2G), and azelaic acid.

Category C: No effectiveness for human pigment spots has been described in scientific journals but may be considered; however, evidence is insufficient. Examples include potassium 4-methoxysalicylic acid and dexpanthenol.

Category D: Not recommended, because of toxicity data described in scientific journals. Examples include Rhododenol, Magnolignan, and ascorbyl tetra-2-hexyldecanoate.

**Table 3:** Effectiveness indices of lightening ingredients developed in Japan.

Effectiveness Indices		Skin-Lightening Ingredients	Test Concentration (%)	General Purpose or Japanese Cosmetics Company	Scientific Articles Providing Evidence
A	Effectiveness of same concentration of cosmetic formulations on human pigment spots has been published in scientific journals and is highly recommended.	tranexamic acid	2	General purpose	[66]
		arbutin	3	General purpose	[31]
		3-O-ethyl ascorbic acid (vitamin C ethyl)	1	General purpose	[8]
		magnesium L-ascorbyl-2-phosphate (APM)	3	General purpose	[2]
		ellagic acid	0.5	General purpose	[36]
		kojic acid	2.5, 0.5	General purpose	[23,24]
		linoleic acid	0.1	General purpose	[52]
		4-n-butyl resorcinol,	0.3	General purpose	[37]
		chamomile extract	0.5	Kao Corporation	[51]
		adenosine monophosphate	3	Otsuka Pharmaceutical Co., Ltd.	[75]
B	Effectiveness of higher concentrations than those used in cosmetics on human pigment spots has been published in scientific journals and is recommended.	oil-soluble licorice extract containing 50% glabridin	0.2	General purpose	[89]
		niacinamide	5, 4	General purpose	[77,78]
		placenta extract	3	General purpose	[17]
		retinol	0.15	General purpose	[90]
		ascorbic acid 2-O-α-glucoside (AA-2G)	20 (iontophoresis)	General purpose	[91]
		azelaic acid.	20	General purpose	[92]
C	No effectiveness for human pigment spots has been published in scientific journals and may be considered, but evidence is insufficient	potassium 4-methoxysalicylate	1, 3	Shiseido Co. Ltd.	
		dexpanthenol		POLA ORBIS HOLDINGS INC.	
D	Not recommended, because of toxicity data published in scientific journals	Rhododenol	2	Kanebo Cosmetics Inc.	[46]
		Magnolignan	0.5	Kanebo Cosmetics Inc.	[46]
		ascorbyl tetra-2-hexyldecanoate		Nikko Chemicals Co. Ltd.	[14,15,16]

The use of a hydrophilic ointment containing 3% APM for hyperpigmentation (melasma and Riehl melanosis) was reported in 1969 [2]. It was reported in 1996 that APM 10% cream was found to be significantly beneficial in 19 of 34 patients with melasma and senile freckles and in 3 of 25 patients with normal skin [4]. The use of 3% human placenta extract for treating facial hyperpigmentation in women (47 cases of melasma and 2 cases of freckles) was reported in 1982 [17]. Other topically applied products with good results for melasma include 1% or 2.5% kojic acid [23], 3% arbutin [31], 1% tranexamic acid [66], 0.1% linoleic acid S [52], 0.3% Rucinol [37], and 0.5% ellagic acid [36]. Examples of topically applied products with good results for senile pigmentation include 3% APM [2], 10% APM [3,4], 1% or 2.5% kojic acid [23,24,25], 0.5% ellagic acid [36], 7% arbutin [87], and 0.5% chamomile extract [51]. Topical application of 1% or 2.5% kojic acid [23], 0.5% ellagic acid [36], 1% tranexamic acid [66], and 7% arbutin [87] has been used with good results in the treatment of freckles. In some cases, 1% vitamin C ethyl has shown effectiveness in pigmentation after natural light exposure and burns [8]. The effectiveness of these pharmaceutical skin-lightening cosmetics was not evaluated in a comparative study with a placebo, but instead was assessed in a study that examined the effectiveness of the formulations before and after continuous use, without double-blinding. In examining effectiveness, attention must be paid to changes in skin tone with seasonal variations and the effects of the base agent.

Examples of topical effectiveness in double-blind comparative studies include 0.1% retinoic acid (Tretinoin) [83], 0.2% oil-soluble licorice extract [89], and 20% azelaic acid [92] for melasma. Topical 0.1% retinol has been reported to be effective in photodamaged skin in a double-blind, controlled study [90]. Iontophoresis of 20% AA-2G has been reported to be effective in melasma and postinflammatory hyperpigmentation [91]. For senile pigmentation, both 0.1% retinoic acid (tretinoin) [84] and 2% 4-methoxyphenol/0.01% retinoic acid [85,86] have shown topical effectiveness in double-blind comparative studies, but these are not active ingredients in pharmaceutical cosmetics. Thus, a pharmaceutical skin-lightening cosmetic product is defined as a pharmaceutical product if its effectiveness against melasma and senile pigmentation is statistically demonstrated in actual use in a double-blind comparison test. 

## 4. Issues in Methods for Evaluation of Skin-Lightening Effects

In Japan, the effectiveness of pharmaceutical skin-lightening quasi-drugs must be statistically demonstrated to prevent or promote the fading of UVB-induced pigmentation, or promote the fading of pigmentation in a UVB-induced pigmentation prevention test or a UVB-induced pigmentation improvement test through double-blind comparison. However, currently different active ingredients are evaluated in various ways to determine their skin-lightening effectiveness. For a quasi-drug to be approved for efficacy, its effectiveness must have been evaluated with an appropriate evaluation method. Moreover, the effectiveness must be properly evaluated with respect to what is used to transmit the efficacy to the consumer. 

Visual evaluation using a skin color chart for more objective and quantitative evaluation is a valuable assessment method [93,94]. For instrumental measurement, the following methods are used: (1) use of a reflectance spectrophotometer (the reflected light is finely spectrophotographed, and the reflectance is measured every 5–10 nm to analyze the color in detail); (2) use of a color chromameter (which measures colors with three sensors—red, blue, and green—close to the sensitivity of the human eye and obtains tristimulus values in the color space); (3) use of a three-wavelength melanin index meter; and (4) analysis of digital images. However, evaluation and analysis methods are advancing rapidly [95,96,97,98]. Importantly, with these methods, data at each time point during the test must be collected under the same conditions for both participants and measurements. Skin color measurements are affected by factors including measurement time, posture, temperature, illumination, and probe pressing force. Therefore, for accurate measurements, nearby normal skin should be used as a control, and the probe pressing force should be kept constant. For images, an illuminance meter or a cast match should be used to confirm that the images are taken at a constant brightness and color.

The most commonly used instrumental measurement is the L*a*b* color system (L*: lightness; a*: chromaticity of red-green coordinates; b*: chromaticity of yellow-blue coordinates) standardized by the Commission Internationale de l’Eclairage (CIE). L* values are often used for skin-lightening evaluation but have the disadvantage of being affected by skin redness [98]. Notably, because the measured value is an average within the range of the measurement area, it cannot be accurately measured if the target area is small. The Mexameter MX18 (Courage + Khazaka, Germany), which measure the melanin index and erythema index by irradiating three wavelengths of light from a probe and measuring the light reflected from the skin, has slightly different hemoglobin indexes. It can assess pigmentation more accurately, although it is slightly affected by hemoglobin. Another method involves taking images of the entire face or part of the face with a CCD camera, calculating the tristimulus XYZ values from the values of the three primary colors of light (red, green, blue) at each pixel, and estimating the amount of melanin from the formula relating these values to the amount of melanin and hemoglobin [98]. The imaging method has the advantage of displaying the distribution of melanin over a wide area of the face. In addition, an ultraviolet camera can capture images of “hidden” pigmentation spots that cannot be recognized by the naked eye [97]. If quantitative measurement and analysis methods are used, this technology may be applied to skin-lightening evaluation. In vivo confocal microscopy can be used to obtain images of melanin granules by using the reflection of light of a certain wavelength, and the location of melanin granules can be determined on the basis of the brightness of the images [99]. In the future, we expect that analysis of skin-lightening effects will advance beyond indirect measurements of light reflected from the skin to noninvasive quantitative measurements of melanin in the skin in vivo.

Because instrumental measurement technology for skin surface conditions and color is continually advancing, and guidelines have been published for evaluating methods of skin-lightening and pigment spot improvement in humans [100], objective evaluation is required not only for pharmaceutical applications but also for those used to communicate efficacy to consumers. In addition, guidelines should be established for in vitro evaluation methods to ensure the appropriateness of methods for measuring inhibition of tyrosinase activity, melanin production and cytotoxicity in cultured cells, because the results differ depending on the evaluation method [41,44,46,101].

## 5. Future Development of Skin-Lightening Ingredients

Because the sale of cosmetic ingredients and cosmetics that have been tested on animals is prohibited, the skin-lightening effect of existing cosmetic ingredients must be demonstrated in clinical trials. Furthermore, skin-lightening ingredient that have high efficacy but cannot be monopolized to increase corporate profits are simply not used as active ingredients in quasi-drugs, because investments are not made in their development. Exfoliating ingredients and parabens currently used in cosmetics are candidates. Exfoliating ingredients, such as glycolic acid and lactic acid, that are used in cosmetics have been demonstrated to lighten skin by removing melanin-deposited stratum corneum [102,103], thus demonstrating that cosmetic ingredients can also lighten skin sufficiently. Parabens, which are commonly used as preservatives in cosmetics and foods, inhibit melanin production more effectively than the active ingredients used in quasi-drugs to lighten skin [104]. Several common cosmetic ingredients can also lighten skin; therefore, the effect of the active ingredients in quasi-drugs must be greater than the skin-lightening effect of cosmetic ingredients, excluding those in makeup cosmetics. Japanese pharmaceutical skin-lightening cosmetics must have “a mitigating effect on freckles and spots,” within the scope of efficacy of quasi-drugs. Current quasi-drugs suppress melanin production and prevent freckles and dark spots. Large melanosome complexes are increased in keratinocytes of solar lentigo [105]. Epidermal turnover time is involved in melanin disappearance [106]. Adenosine monophosphate and dexpanthenol, owing to their mechanisms of action, effectively promotes melanin excretion from the epidermis by promoting epidermal turnover. In line with the actual use scenarios of skin-lightening cosmetics by consumers, who expect their spots and freckles to fade and disappear [88], a specialist committee for evaluation of skin-lightening effectiveness of the Japanese Cosmetic Science Society has proposed “gentle improvement of spot and freckle pigmentation” as a new efficacy standard [100]. The proposal was made at a meeting of the cosmetic functional evaluation methodology review committee of the Japan Cosmetic Science Society. Moreover, to avoid problems with leukoderma, in Japan, as in other countries, third-party test results, rather than the cosmetics company’s own test results, should be evaluated for approval. The definition of skin-lightening, efficacy, and evaluation methods will be better suited to consumer needs through academic collaboration among universities, dermatologists, and companies in the future.

## 6. Conclusions

In Japan, approximately 20 active ingredients for skin brightening have been developed and approved, and sales of pharmaceutical skin lightening cosmetics containing these ingredients have also been approved. Some treatments have no apparent effect on spots and freckles, cause leukoderma, or cause allergic reactions. One of the reasons for these problems may be that the results of in-house effectiveness and safety tests have not been publicly reviewed. Objective evaluation by a third-party organization is needed to solve these problems. Because the sale of cosmetic ingredients and cosmetics that have been tested on animals is prohibited, pharmaceutical skin-lightening cosmetics containing active ingredients that have been demonstrated to be effective in clinical trials on existing cosmetic ingredients are expected to be marketed in the future.

## Figures and Tables

**Table 1:** List of skin-lightening active ingredients approved in Japan.

Approved Year	Generic Name	Development Company	Chemical Name/Substance Name	Main Mechanism of Action
	placenta extract			
1983	magnesium ascorbyl phosphate (APM)	Takeda Pharmaceutical Co., Ltd.	magnesium L-ascorbyl-2-phosphate	tyrosinase inhibition
1988	kojic acid	Sansho Seiyaku Co., Ltd.	kojic acid	tyrosinase inhibition
1989	arbutin	Shiseido Co., Ltd.	hydroquinone-β-D-glucopyranoside	tyrosinase inhibition
1994	ascorbyl glucoside (AA-2G)	Hayashibara Co., Ltd., Kaminomoto Co., Ltd., Shiseido Co., Ltd.	L- ascorbic acid 2-O-α-glucoside	tyrosinase inhibition
1997	ellagic acid	Lion Corporation	ellagic acid	tyrosinase inhibition
1998	Rucinol^®^	Kurarey Co., Ltd. POLA Chemical industries, Inc.	4-n-butylresorcinol	tyrosinase inhibition
1999	Chamomile ET	Kao Corporation	*Matricaria chamomilla* L Extract	endothelin blocker
2001	linoleic acid S	Sunstar Inc.	linoleic acid	tyrosinase degradation, stimulation of epidermal turn over
2002	tranexamic acid (t-AMCHA)	Shiseido Co., Ltd.	trans-4-aminocyclohexane carboxylic acid	inhibition of prostaglandin E_2_ production by anti-plasmin
2003	4MSK	Shiseido Co., Ltd.	potassium 4-methoxysalicylate	tyrosinase inhibition
2004	Vitamin C ethyl	Nippon Hypox Laboratories, Inc.	3-O-ethyl ascorbic acid	tyrosinase inhibition
2004	Energy signal AMP^®^	Otsuka Pharmaceutical Co., Ltd.	adenosine mono phosphate	stimulation of epidermal turnover
2005	Magnolignan^®^	Kanebo Cosmetics Inc.	5,5-dipropyl-biphenyl-2,2-diol	inhibition of tyrosinase maturation, cytotoxicity to melanocytes
2007	D-Melano (niacinamide W)	P&G Maxfactor	niacinamide	suppression of melanosome transfer
2008	Rhododenol^®^	Kanebo Cosmetics Inc.	4-(4-hydroxyphenyl)-2-butanol, Rhododendrol	tyrosinase inhibition, cytotoxicity of melanocytes
2008	TXC	CHANEL	tranexamic acid cetyl ester hydrochloride	inhibition of prostaglandin E_2_ production
2009	ascorbyl tetraisopalmitate	Nikko Chemicals Co., Ltd.	ascorbyl tetra-2-hexyldecanoate	tyrosinase inhibition
2018	dexpanthenol W (PCE–DP)	POLA ORBIS Holdings Inc.	dexpanthenol	enhance energy production of epidermal cells

## Data Availability

Not applicable.
